# Fibrosis-4 Score and Postoperative Outcomes in Metabolic Dysfunction-Associated Steatotic Liver Disease

**DOI:** 10.5152/tjg.2026.25693

**Published:** 2026-01-05

**Authors:** Fatih Eren, Mehmet Refik Goktug, Derya Ari, Gulsah Fidan Ozkumur, Eda Nur Bulbuller, Genco Gencdal, Yuksel Guleryuzlu, Caglayan Keklikkiran, Dinc Dincer, Dilara Turan Gokce, Askin Erdogan, Meral Akdogan Kayhan, Mehmet Kursad Keskin, Selcan Akesen, Gokhan Ocakoglu, Murat Kiyici

**Affiliations:** 1 Department of Gastroenterology, Bursa Uludağ University School of Medicine, Bursa, Türkiye; 2 Department of Gastroenterology, Ankara Bilkent City Hospital, Ankara, Türkiye; 3 Department of Gastroenterology, Akdeniz University School of Medicine, Antalya, Türkiye; 4 Department of Internal Medicine, Alaaddin Keykubat University, Antalya, Türkiye; 5 Department of Gastroenterology and Hepatology, Koç University School of Medicine, İstanbul, Türkiye; 6 Department of Gastroenterology, Kartal Dr. Lutfi Kırdar City Hospital, İstanbul, Türkiye; 7 Department of Gastroenterology, Recep Tayyip Erdogan University School of Medicine, Rize, Türkiye; 8 Department of Gastroenterology, Ankara Sincan Training and Research Hospital, Ankara, Türkiye; 9 Department of Gastroenterology, Alaaddin Keykubat University, Antalya, Türkiye; 10 Department of Anaesthesiology and Reanimation, Bursa Uludağ University School of Medicine, Bursa, Türkiye; 11 Department of Biostatistics, Bursa Uludağ University School of Medicine, Bursa, Türkiye

**Keywords:** Adverse events, FIB-4 score, metabolic dysfunction-associated steatotic liver disease, mortality

## Abstract

**Background/Aims:**

The prevalence of metabolic dysfunction-associated steatotic liver disease (MASLD) is increasing globally. The Fibrosis-4 (FIB-4) score is a noninvasive biomarker used for assessing potential advanced fibrosis. The study aimed to evaluate the role of the FIB-4 score in predicting postoperative complications and mortality in patients undergoing surgery.

**Materials and Methods:**

This multicenter retrospective study included 11 072 patients who underwent surgery under general anesthesia. Demographic and clinical data—including age, gender, comorbidities, FIB-4 scores, American Society of Anesthesiologists classification, postoperative complications, and mortality—were analyzed.

**Results:**

A total of 1667 MASLD patients were included. Patients were classified based on FIB-4 scores: 70% (n = 1167) had FIB-4 < 1.30, 25.1% (n = 418) had 1.30 < FIB-4 ≤ 2.67, and 4.9% (n = 82) had FIB-4 ≥ 2.67. Due to the limited number of patients with possible advanced fibrosis (FIB-4 ≥ 2.67), propensity score (PS) matching was performed. After PS matching, patients with a high FIB-4 score exhibited a significantly higher rate of postoperative complications (*P* < .001), and 12-month mortality was elevated (11%), although the difference was not statistically significant (*P* = .481).

**Conclusion:**

A high FIB-4 score may serve as a predictive marker for postoperative complications in patients with MASLD undergoing surgery.

**Main Points**

- Metabolic dysfunction-associated steatotic liver disease (MASLD) has become increasingly prevalent worldwide and is associated with higher rates of postoperative complications.
- A high Fibrosis-4 (FIB-4) score (FIB-4 ≥ 2.67) is associated with an increased risk of postoperative complications.
- The FIB-4 score may serve as a useful tool for preoperative risk stratification in patients with MASLD.

## Introduction

The incidence of metabolic dysfunction-associated steatotic liver disease (MASLD), formerly known as nonalcoholic fatty liver disease, is rising worldwide, with an estimated prevalence of 25%-30% among adults.^1^ The MASLD is characterized by macrovesicular steatosis affecting more than 5% of hepatocytes in the absence of significant inflammation or fibrosis.^2^ Key metabolic risk factors include obesity (BMI ≥25 kg/m^2^), hypertension (HT), dyslipidemia, and type 2 diabetes mellitus (DM). Other potential causes of fatty liver, such as medication use and prolonged fasting, should be excluded, and alcohol consumption must be minimal (<20 g/day in women and <30 g/day in men).^3^

Liver fibrosis is the most critical determinant of long-term clinical outcomes in MASLD. While liver biopsy remains the gold standard for fibrosis assessment, it is invasive and carries procedural risks.^4^ Consequently, non-invasive, cost-effective alternatives have been explored to estimate the presence and severity of hepatic fibrosis. Several imaging techniques and noninvasive methods based on serum biochemical tests have been developed to assess liver fibrosis.^5^ Two widely used noninvasive imaging techniques are shear wave elastography and vibration-controlled transient elastography (VCTE), which provide high accuracy in evaluating fibrosis by measuring liver stiffness.^6^ However, their availability remains limited. Consequently, biochemical test-based noninvasive methods have gained prominence. The Clinical Practice Guidelines recommend the Fibrosis-4 (FIB-4) score as a simple, effective tool for ruling out advanced fibrosis.^7^

The FIB-4 score is a noninvasive, widely available, and cost-effective tool commonly used in clinical practice. It can be calculated using only the patient’s age, aspartate aminotransferase (AST), alanine aminotransferase (ALT), and platelet count.^8^ The MRI-based assessments of liver fibrosis and steatosis offer a potential alternative to liver biopsy in clinical settings. However, in clinical practice, the FIB-4 score is an accurate tool and demonstrates diagnostic performance comparable to magnetic resonance elastography to predict advanced liver fibrosis.^9^

Multiple studies have demonstrated that MASLD, particularly in its advanced stages, is associated with an increased risk of all-cause mortality.^10^ The primary causes of mortality include cardiovascular diseases (CVDs), extrahepatic malignancies, and liver-related complications.^11^ Additionally, liver diseases heighten the risk of surgical and anesthetic complications. The severity of liver disease is a key determinant of perioperative morbidity and mortality.^12^ A recent study of patients undergoing general anesthesia found that a high FIB-4 score correlated with increased mortality.^13^ Other studies have also identified MASLD severity in metabolic syndrome patients as an independent risk factor for postoperative complications.^14^

This study aimed to assess the diagnostic accuracy of the FIB-4 score in predicting adverse postoperative outcomes in patients with MASLD.

## Materials and Methods

### Study Population

**Figure 1.**
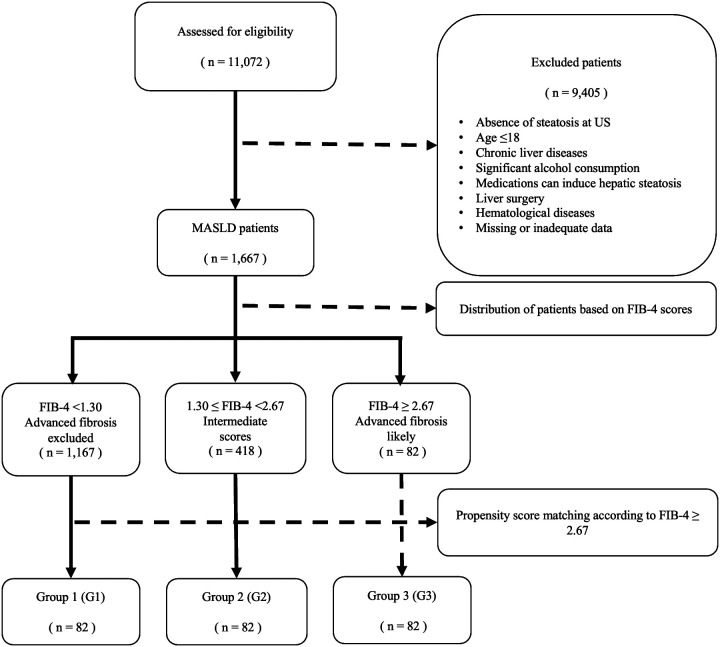
The patient selection process based on inclusion and exclusion criteria.

This multicenter study involved 7 tertiary care centers in Türkiye. The study retrospectively analyzed 11 072 patients aged ≥18 years who underwent general anesthesia for surgical procedures between September 2012 and 2022. The collected data included age, gender, height, weight, body mass index (BMI), ultrasonographic findings, DM, HT, CVDs, AST, ALT, platelet count, preoperative American Society of Anesthesiology (ASA) physical status scores, postoperative complications, and mortality rates. Figure 1 illustrates the patient selection process based on inclusion and exclusion criteria.

Patients were categorized into 3 groups based on FIB-4 score: group 1 (G1): ≤1.3 (low likelihood of advanced fibrosis), group 2 (G2): 1.3-2.66 (intermediate risk), and group 3 (G3) ≥2.67 (high likelihood of advanced fibrosis).

The ASA Physical Status Classification is as follows: ASA-PS I, normal healthy patient; ASA-PS II, patient with mild systemic disease; ASA-PS III, patient with severe systemic disease; ASA-PS IV, patient with severe systemic disease that is a constant threat to life; ASA-PS V, moribund patient not expected to survive without the operation; and ASA-PS VI, a brain-dead patient undergoing organ donation.

### Inclusion Criteria

Patients with any degree of fatty liver confirmed via ultrasonography and one of the cardiometabolic criteria; (i) BMI ≥ 25 kg/m^2^ or waist circumference >94 cm (for males) or 80 cm (for females); (ii) fasting serum glucose ≥100 mg/dL or 2 hour post-load glucose ≥100 mg/dL or hemoglobin A1c ≥ 5.7 or type 2 DM or treatment for type 2 DM; (iii) blood pressure ≥130/85 mmHg or specific antihypertensive drug treatment; (4) triglycerides ≥150 mg/dL or lipid-lowering medication use; (5) HDL cholesterol <40 mg/dL for males and <50 mg/dL for females or lipid-lowering medication use were included.

To determine the FIB-4 score, only patients with recorded AST, ALT, and platelet counts within 6 months preoperatively were considered. Additionally, patients with at least 12 months of postoperative follow-up were assessed for complications and mortality.

### Exclusion Criteria

Patients with chronic liver disease, cirrhosis, acute and chronic hepatitis, significant alcohol use (>20 g/day for women and >30 g/day for men), alcoholic liver disease, hepatotoxic drug use, Wilson’s disease, hereditary hemochromatosis, autoimmune hepatitis, or prior liver transplantation were excluded. Patients with hematological conditions (immune thrombocytopenia, bone marrow disorders) affecting platelet levels were also excluded, as such conditions could influence FIB-4 score calculations.

### Endpoints

The primary study endpoint was 12-month mortality. Postoperative complications were examined as secondary endpoints.

The complications were classified according to the involved system as surgical site complications (including wound complications and bleeding), cardiovascular, respiratory, infectious, urogenital, and neurological complications. Additionally, postoperative complications were analyzed by categorizing them into 2 groups based on pathogenesis: infectious and non-infectious complications.

### Risk Factor Evaluation Criteria

The FIB-4 score served as the primary biochemical marker of liver fibrosis and was calculated using the formula: FIB-4 = age (year) × AST (U/L)/platelet count (×10^9^/L) × √ALT (U/L).

### Ethics Approval Statement

This study was performed in line with the principles of the Declaration of Helsinki. Approval was granted by the Ethics Committee of Bursa Uludağ University dated December 5 2023, with the number 2023-25/26. Given the retrospective nature of the study, informed consent was waived by the Ethics Committee of Bursa Uludağ University dated December 5 2023, with the number 2023-25/26.

### Statistical Analysis

Propensity score (PS) matching was employed to mitigate confounding effects due to baseline characteristic imbalances across FIB-4 categories. A PS for each patient was calculated using multivariable logistic regression, factoring in age and sex, to estimate the probability of belonging to the FIB-4 ≥2.67 group. This analysis compared patients across 2 groups: those with FIB-4 <1.30 and those with 1.30 < FIB-4 < 2.67.

The Hosmer–Lemeshow test was performed to assess the goodness of fit for the multivariable logistic regression model. To minimize confounding effects from baseline characteristic imbalances, nonparametric nearest neighbor PS matching was performed using NCSS 2019 statistical software (NCSS; Kaysville, USA). A 1 : 1 : 1 matching ratio was used, aligning patients in the FIB-4 ≥2.67 group with those in the FIB-4 <1.30 and 1.30 < FIB-4 < 2.67 groups based on age and sex.

The Shapiro–Wilk test was used to determine the normality of age distribution, and results were reported as means and standard deviations. ANOVA was applied for group comparisons. Categorical variables were analyzed using chi-square and Fisher–Freeman–Halton tests.

Post-hoc subgroup analyses were conducted with Bonferroni correction, and adjusted *P* values were reported. Logistic regression analysis was performed to identify factors influencing mortality. All statistical analyses were conducted using IBM SPSS Statistics for Windows, Version 25.0 (IBM Corp., Armonk, New York), with a significance threshold of *P* < .05.

## Results

### Baseline Characteristics

After applying inclusion and exclusion criteria, 1667 patients were included in the study (899 females (53.9%) and 768 males (46.1%); mean age: 58 years (range: 19-92)). The mean BMI was 29.03 (range: 13-54). The most prevalent comorbidity was HT (30.5%), followed by DM (20.7%) and CVDs (10.5%). The baseline characteristics of the study population are listed in Table 1. Among the 777 patients with known ASA classification, 24.3% were ASA I, 63.2% were ASA II, 12.2% were ASA III, and 0.3% were ASA IV. The mean FIB-4 score was 0.97 (range: 0.12–16.26). Patients were stratified as follows: FIB-4 <1.30 (n = 1167, 70.0%), 1.30 < FIB-4 ≤ 2.67 (n = 418, 25.1%), and FIB-4 ≥2.67 (n = 82, 4.9%). Due to the small number of patients in the FIB-4 ≥2.67 group, PS matching was applied.

### Postoperative Complications

Surgical site complications were the most common postoperative complications in the study groups. Gastrointestinal complications were the second most common in the FIB-4 ≥2.67 group. The FIB-4 ≤1.3 group had the lowest general complication rate of 4.45%. Furthermore, in the overall study cohort, infectious complication rates were 1.97% in the FIB-4 <1.30 group, 12.68% in the 1.30 < FIB-4 ≤ 2.67 group, and 10.98% in the FIB-4 ≥2.67 group. Table 2 presents the distribution of complications in the overall study cohort, categorized by the involved system prior to PS matching.

### Propensity Score Matching Results

Following PS matching, no significant differences were observed between the 3 groups regarding gender distribution (*P* = .937) and age (*P* = .995). However, DM prevalence varied significantly among groups (*P* = .004), with rates of 12.2% in G1 (FIB-4 <1.30), 22% in G2 (1.30 < FIB-4 ≤ 2.67), and 34.1% in G3 (FIB-4 ≥2.67). A significant difference was found between G1 and G3 groups (*P* < .05), indicating a higher DM prevalence in patients with advanced fibrosis. No significant differences were noted for HT, CVDs, or other comorbidities (*P* = .061, *P* = .441, and *P* = .763, respectively). Additionally, ASA classification did not significantly differ among groups (*P* = .368).

Postoperative complications were significantly more frequent in the advanced fibrosis group (*P* < .001). No complications were reported in G1, while complication rates were 9.5% in G2 and 21.9% in G3. A significant difference was observed between G3 and the other groups (*P* < .005). Mortality rates were 6.9% in G1, 6.1% in G2, and 11% in G3, but the difference was not statistically significant (*P* = .481). Table 3 presents the demographic characteristics and group comparisons.

### Logistic Regression Analysis for Mortality Risk

To determine mortality risk factors, univariate logistic regression analysis was first conducted for variables listed in Table 4. Variables with *P* < .25 were included in the multivariate logistic regression model, identifying BMI, ASA score, HT, and CVDs as potential predictors. The final model demonstrated a good fit (*P* = .823); however, none of the included variables significantly increased mortality risk.

## Discussion

Current clinical practice guidelines recommend the use of the FIB-4 score to assess fibrosis risk in patients with MASLD. This study evaluated the utility of the FIB-4 score in the context of preoperative risk assessment. The findings suggest that the FIB-4 index may help identify the risk of postoperative complications in MASLD patients with suspected advanced fibrosis. However, no statistically significant difference in mortality was observed.

A study by Zelber-Sagi et al,^13^ involving 19 861 patients without overt liver disease in which patients from a similar age group to the cohort were evaluated, demonstrated that FIB-4 ≥2.67 was significantly associated with increased intraoperative complications, longer hospitalization, and higher 30-day postoperative mortality (0.1%, 1.4%, and 2.1%, respectively) compared with FIB-4 <1.30. Similarly, MASLD has been identified as a major risk factor for postoperative complications and liver failure following liver resection.^15^ In patients undergoing hepatectomy for colorectal cancer liver metastases, Akiyama et al^16^ reported significantly shorter overall survival in those with high FIB-4 >2.736. Another study on gastric cancer patients undergoing gastrectomy found that high FIB-4 scores correlated with shorter overall survival.^17^ These findings align with the study, where mortality rates were higher with FIB-4 ≥2.6, although the difference was not statistically significant.

The ASA classification remains the most widely used tool for preoperative risk evaluation. One study reported mortality rates of 0.02%, 0.14%, and 1.41% for ASA-PS I, II, and III patients, respectively, which surged to 11.14% in ASA IV patients.^18^ Large-scale analyses have shown an 89-fold increase in mortality risk among ASA IV patients.^19^ However, all MASLD patients in the propensity-matched cohort were ASA-PS I–III, and no significant difference in mortality and postoperative complications was observed among the 3 groups. The ASA physical status classification system is a simple tool, but it has limitations. One of its limitations is that the ASA classification does not include an important parameter that may affect perioperative risk, such as age. The risks of general anesthesia and surgery are higher in elderly patients than in younger patients.^20^ Additionally, the severity of existing comorbidities varies among patients and may have different effects on postoperative outcomes.^21^ Another limitation is that the ASA classification system shows inter-rater variability. In a study, the ASA score was evaluated among medical experts in hepato-pancreato-biliary surgery multicenters. An online survey including 8 HPB cases, general questions, and considerations of the ASA score was delivered to the members of international societies. The survey showed a significant inter–rater variability in applying and interpreting the ASA score. They determined that the survey showed the need to improve the ASA score because there is an absence of consensus.^22^ The degree of fibrosis in MASLD patients affects postoperative adverse outcomes. The advantage of the FIB-4 score is that it is a standardized method and does not show inter-rater variability. The FIB-4 score may have utility and be more objective in clinical decision-making. Therefore, the FIB-4 score can be used in preoperative risk assessment in MASLD patients, in addition to the ASA physical status classification system.

High FIB-4 scores have been associated with an increased risk of postoperative complications. In a study evaluating patients who underwent esophagectomy for esophageal cancer, those with FIB-4 scores >1.44 had a 3.78-fold higher risk of anastomotic leakage.^23^ Similarly, Dong et al^24^ identified a FIB-4 cutoff score of 2.88 for predicting postoperative complications and 3.85 for intraoperative blood loss in patients with hepatocellular carcinoma undergoing hepatectomy. Consistent with these findings, the study demonstrated a significantly higher incidence of postoperative complications in patients with FIB-4 scores ≥2.67. Increased postoperative complications in patients with MASLD may be due to increased systemic inflammation. MASLD is not only an important cause of progressive liver disease but also a chronic inflammatory condition that causes significant systemic effects. The most important of these is that it causes subclinical and clinical CVD. The liver contains innate immune cells such as macrophages and dendritic cells and has important functions in the systemic inflammatory response. These cells activate the innate immune response in the liver and trigger inflammation.^25^ Systemic inflammatory markers such as interleukin-6 (IL-6) and tumor necrosis factor alpha have been found to be elevated in the serum of patients with MASLD and are considered responsible for cardiovascular effects.^13^^,^^26^ Furthermore, studies have found that high postoperative IL-6 levels are associated with increased postoperative complications in patients undergoing major abdominal surgery.^27^ The IL-6 levels increase as MASLD progresses and have been shown to correlate with disease severity.^28^ The increased postoperative complications in the group with advanced fibrosis may be related to the increased inflammatory status in this group of patients.

Diabetes mellitus may independently contribute to fibrosis progression. In a study by Yongsheng et al^29^ involving patients who underwent bariatric surgery, the prevalence of MASLD was 68.9%. Among these patients, 9.1% had significant fibrosis, and 4.0% had advanced fibrosis, with the FIB-4 score effectively identifying fibrosis risk. In the study, a higher incidence of significant fibrosis was observed in patients with DM compared to those without DM. Advanced age and DM are identified as risk factors for fibrosis progression.^30^ In another study, the ultrasonographic prevalence of hepatic steatosis in 6283 patients with type 2 DM was 69.9%, approximately one-quarter had a FIB-4 ≥1.3, and referral to hepatology/gastroenterology remained low (15.5%).^31^ Additionally, a study on liver transplantation found that patients with DM had higher FIB-4 scores, suggesting a greater likelihood of fibrosis development post-transplantation.^32^ The study similarly found that DM was significantly more prevalent in patients with elevated FIB-4 scores, further supporting its role as an independent risk factor for fibrosis.

A key strength of this study is its focus on the direct relationship between FIB-4 scores and postoperative outcomes specifically in MASLD patients—an area with limited existing research. The large sample size and multicenter design enhance the study’s generalizability. Furthermore, the standardized FIB-4 calculation method and well-defined endpoints minimize the risk of confounding. Due to the relatively small number of patients with FIB-4 scores ≥2.67, PS matching was applied to minimize bias, ensuring comparability between groups.

However, the retrospective nature of the study presents limitations, including missing data that necessitated patient exclusion. The VCTE findings could not be incorporated into the analysis due to the unavailability of VCTE technology across all participating centers. In addition, ultrasonography has limited sensitivity in patients with mild steatosis; therefore, patients with mild steatosis may have been excluded from the study. The type of surgery and the experience of the surgeon, which may affect postoperative complications, were quite heterogeneous. Methods that could predict postoperative outcomes, such as the Charlson Comorbidity Index and ACS-NSQIP Surgical Risk scoring systems, were not applied to most of the patients and data were lacking, so comparisons with these scoring systems could not be made. Future prospective studies are needed to adequately assess these parameters.

A high FIB-4 score is a significant predictor of postoperative adverse outcomes in MASLD patients. Given the elevated risk of postoperative complications in this population, the FIB-4 score should be considered a valuable tool for preoperative risk assessment.

## Figures and Tables

**Table 1.** Baseline Characteristics of the Study Population

**Parameters**	**Mean (Range)/n(%)**
Age (years)	58 (19-92)
Sex	
Female	899 (53.9)
Male	768 (46.1)
BMI	29.03 (13-54)
Comorbidities	
DM	345 (20.7)
HT	508 (30.5)
CVDs	175 (10.5)
Other comorbidities	683 (40.9)
Preop FIB-4	
≤1.30	1167 (70.01)
1.30-2.67	418 (25.07)
≥2.67	82 (4.92)
AST (U/L)	19 (2-55)
ALT (U/L)	20 (3-881)
Platelets (G/L)	255 (25-870)
ASA-PS I	189 (24.3)
ASA-PS II	491 (63.2)
ASA-PS III	82 (4.9)

ALT, alanine aminotransferase; AST, aspartate aminotransferase; ASA-PS, The American Society of Anesthesiologists Physical Status; BMI, body mass index; CVD, cardiovascular disease; DM, diabetes mellitus; HT, Hypertension.

**Table 2.** Distribution of Complications According to the Involved Systems before PS matching

Complications	**FIB-4 ≤1.3** **(n = 1167)**	**1.30< FIB-4 <2.67** **(n = 418)**	**FIB-4 ≥2.67** **(n = 82)**
Surgical site Wound infection Suture opening Hematoma Abscess	21 (1.80%) 13 0 7 1	48 (11.48%) 43 2 1 2	7 (8.53%) 6 1 0 0
Cardiovascular Cardiac arrhythmia Myocardial infarction	5 (0.43%) 5 0	14 (3.35%) 13 1	3 (3.66%) 3 0
Respiratory Pulmonary atelectasis Pulmonary edema Pneumonia Pulmonary embolism	13 (1.11%) 5 4 4 0	15 (3.59%) 4 3 4 4	1 (1.22%) 0 1 0 0
Infections Sepsis	5 (0.43%) 5	4 (0.96%) 4	3 (3.66%) 3
Urogenital Acute kidney injury	0 0	1 (0.24%) 1	0 0
Gastrointestinal Ileus Pancreatitis	7 (0.59%) 5 2	3 (0.71%) 3 0	4 (4.88%) 4 0
Neurologic Stroke Seizure	1 (0.09%) 1 0	1 (0.24%) 0 1	0 0 0
Total number	52 (4.45%)	86 (20.57%)	18 (21.95%)

FIB-4, Fibrosis-4; PS, propensity score.

**Table 3.** Comparison of Demographic Characteristics and Comorbidities Between Study Groups After PS Matching

**Parameter**	**Before PS Matching**	**After PS Matching**			*P*
		**FIB-4 <1.30 ** **(n = 82)**	**1.30<FIB-4 <2.67** **(n = 82)**	**FIB-4 ≥2.67** **(n = 82)**	
Gender					.937
Female	899 (53.9%)	41 (50%)	43 (53.4%)	41 (50%)	
Male	768 (46.1%)	41 (50%)	39 (47.6%)	41 (50%)	
Age (years)	58 (19-92)	58.80 ± 13.64	58.60 ± 13.67	58.78 ± 13.60	.995
Diabetes mellitus	345 (20.7%)	10 (12.2%)	18 (22%)	28 (34.1%)	**.004**
Hypertension	508 (30.5%)	19 (23.2%)	31 (38.3%)	31 (38.3%)	.061
Cardiovascular disease	175 (10.5%)	9 (11%)	14 (17.3%)	14 (17.1%)	.441
Other comorbidities	683 (40.9%)	34 (60.7%)	40 (64.5%)	41 (67.2%)	.763
ASA-PS classifications (n = 777)					.368
ASA-PS I	189 (24.3%)	13 (29.5%)	8 (24.2%)	5 (19.2%)	
ASA-PS II	491 (63.2%)	26 (59.1%)	16 (48.5%)	17 (65.4%)	
ASA-PS III	82 (4.9%)	5 (11.4%)	9 (27.3%)	4 (5.4%)	
Postoperative complications	97 (5.81%)	0	6 (9.5%)	14 (21.9%)	**<.001**
Mortality	52 (3.12%)	4 (6.9%)	5 (6.1%)	9 (11%)	.481

Data are expressed as mean ± standard deviation and n (%).

ASA-PS, The American Society of Anesthesiologists Physical Status; ASMD, absolute standardized mean difference; BMI, body mass index; FIB-4, fibrosis-4; PS, propensity score.

Bold values indicate statistically significant results (*P* < .05).

**Table 4.** Factors Affecting Mortality

	**Univariate Logistic ** **Regression Model**		**Multivariate Logistic ** **Regression Model**	
	**Crude OR (95% CI)**	*P*	**Adjusted OR (95% CI)**	*P*
Gender (male)	1.38 (0.52-3.64)	.516		
Age (years)	0.98 (0.95-1.02)	.282		
BMI (kg/m^2^)	0.85 (0.74-0.99)	**.031**	0.87 (0.66-1.15)	.870
ASA-PS	2.93 (0.76-11.33)	**.120**	4.38 (0.63-30.71)	.137
DM	0.84 (0.26-2.65)	.760		
HT	2.36 (0.89-6.24)	**.084**	2.82 (0.35-23.02)	.334
CVDs	2.13 (0.71-6.41)	**.177**	0.99 (0->100)	.998
AST	1.01 (0.97-1.02)	.566		
ALT	1.01 (0.99-1.02)	.933		
Platelet count	0.99 (0.98-1.01)	.411		
FIB-4				
FIB-4 <1.30	1	–		
1.30 <FIB-4 ≥ 2.67	0.88 (0.23-3.42)	.849		
FIB-4 ≥2.67	1.66 (0.49-5.69)	.417		

ALT, alanine aminotransferase; AST, aspartate aminotransferase; BMI, body mass index; CVD, cardiovascular diseases; DM, diabetes mellitus; HT, hypertension; OR, odds ratio.

## Data Availability

The data that support the findings of this study are available on request from the corresponding author.
